# Zwitterionic Polymer Gel Fracturing Fluid with Molecular Interface Regulation for Pretreatment-Free Flowback Recycling

**DOI:** 10.3390/gels12020178

**Published:** 2026-02-19

**Authors:** Qingguo Wang, Cuilong Kong, Zhixuan Zhu, Guang Shi, Xuesong Lin, Shengnan Shi, Silong Gai, Jianxun Meng

**Affiliations:** 1 Daqing Oilfield Production Technology Institute, Daqing 163453, China; 2 Heilongjiang Provincial Key Laboratory of Oil and Gas Reservoir Stimulation, Daqing 163453, China; 3 College of Chemistry and Chemical Engineering, Northeast Petroleum University, Daqing 163318, China

**Keywords:** flowback fluid recycling, molecular interface regulation, fracturing fluid, salt-resistant polymer, zwitterionic TMAO

## Abstract

High salinity and hardness in flowback fluids from tight reservoirs severely degrade the performance of conventional fracturing fluids, leading to formation damage and imposing major constraints on water recycling. An innovative in situ molecular interface regulation strategy that bypasses the need for costly pretreatment was proposed. A novel zwitterionic polymer was synthesized by grafting trimethylamine N-oxide (TMAO) onto hydrolyzed polyacrylamide. This hydrolyzed polyacrylamide grafted with trimethylamine N-oxide polymer (HPAMT) leverages zwitterionic TMAO groups to form a robust hydration layer approximately 0.25 nm thick on the polymer chains. Each TMAO group can immobilize up to 22.2 water molecules, effectively shielding the polymer from the detrimental effects of ions like Ca^2+^ and Na^+^, thereby preventing chain curling and preserving cross-linking sites. Experimental results demonstrate that HPAMT fracturing fluid prepared with untreated flowback fluids retains over 70% of its initial viscosity. The HPAMT fracturing fluid exhibits superior thermal and shear stability, maintaining more than 90% viscosity after exposure to 90 °C and the shear rate of 170 s^−1^ for 60 min. Furthermore, HPAMT provides excellent proppant suspension, exceeding 60 min of static settling time. The broken gel viscosity remains below 5 mPa·s, enabling the direct reuse of flowback water. This technology overcomes the critical compatibility issue between traditional polymers and challenging brine chemistry, significantly reducing freshwater consumption and operational costs, thus presenting a viable and innovative solution for enhancing the environmental sustainability of unconventional resource development.

## 1. Introduction

The development of global unconventional oil and gas resources heavily relies on hydraulic fracturing technology [1,2,3]. A single fracturing operation per well can consume approximately 7600 to 19,000 cubic meters of freshwater (equivalent to 2 to 5 million gallons), approximately 3 to 5 times the regional water resource carrying capacity, further exacerbating water scarcity issues [4]. During fracturing operations, fracturing fluids are injected into the reservoir through wellbore piping. After treatment of fracturing and gel-breaking, fracturing fluids flow back from the wellbore to form flowback fluid. Flowback fluid typically contains high-salinity salts, whose total dissolved solids exceeds 20,000 mg/L, divalent metal ions including calcium and magnesium with the concentration over 5000 mg/L, and residual chemical additives such as cross-linkers and gel breakers. Direct discharge of fracturing flowback fluid may cause soil salinization and groundwater contamination, posing serious threats to the environment. Although advanced treatment of flowback fluid can mitigate environmental risks, the cost of this process accounts for over 35% of the total fracturing investment, resulting in poor economic efficiency [5,6,7].

Direct recycling of flowback fluid has become a current research topic to resolve the predicament formed by water consumption, pollution control, and economic cost [8,9,10]. Theoretically, the reuse of flowback fluid can achieve a freshwater substitution rate of 90% and reduce wastewater treatment costs by 70%. However, the synergistic inhibitory effects between flowback fluid components and fracturing fluid performance remain a bottleneck for technical application. Calcium and magnesium ions competitively occupy the cis-hydroxyl cross-linking sites on guar gum molecules, leading to a significant decrease in gel strength. Residual oxidizers from ammonium persulfate-type gel breakers may cause main-chain scission in hydrophobically associating polymers. Under high Chemical Oxygen Demand (COD) conditions (>8000 mg/L), microbial proliferation may exacerbate reservoir pore throat plugging and damage. More severely, flowback fluid retention in the reservoir can cause over 50% permeability damage, primarily due to liquid phase retention, solid residue blockage, and salt crystallization effects [11].

Despite having made progress, physical and chemical methods for flowback fluid treatment still exhibit significant limitations so far. Multi-stage filtration and membrane separation processes can reduce suspended solid concentration below 5.0 mg/L; however, the energy consumption exceeds 15.0 kWh/m^3,^ and the interference of dissolved ions on fracturing fluid crosslinking cannot be eliminated. The technology, combined with coagulation and adsorption, can improve COD removal rates, while it has limited efficiency for high-valent metal ion removal and struggles to meet large-scale treatment demands [12]. Though chemical heat-generating energization technology can enhance fracturing fluid flowback rates by releasing heat and gas, its acidic condition requirements have poor compatibility with existing fracturing fluids, posing risks to wellbore integrity. The lengthy treatment cycle (3–5 days) of microbial degradation technology, while effective for organic content reduction, hinders its application in scenarios requiring continuous operation. Low-temperature gel breaker activator treatment for flowback fluid can improve gel-breaking efficiency by reducing reaction activation energy, but the reaction is violent with poor time controllability, easily causing premature gel breaking before the proppant fully spreads. Furthermore, changes in the solid particle size distribution within flowback fluid directly affect the degree of reservoir damage, decrease in the particle size of the solid phase can lead to easier blockage of micro-cracks and micro-fractures, thereby exacerbating permeability damage [13]. Therefore, employing molecular design strategies to optimize the interfacial behavior between contaminants and fracturing fluid, circumventing traditional advanced treatment, has become a promising solution. Hydrophobically associating polymers based on molecular design maintain viscosity in high-salinity environments due to the hydrophobic microdomains, while residual persulfate-induced chain scission reactions reduce their molecular weight by over 50%, with viscosity retention below 35% at 120 °C. The molecular structure of zwitterionic polymers has inhibited calcium ion complexation with guar gum hydroxyl groups in the fracturing fluid by electrostatic shielding effects, increasing viscosity retention to 80%. However, the protection against oxidizer degradation is always insufficient [14,15]. Despite the dual promise of preserving fracturing fluid performance and enabling flowback fluid reuse, in situ molecular interface regulation has seen only marginal improvement in recycling efficiency. Parallel developments in molecular design across related disciplines have highlighted instructive structural motifs. The zwitterionic osmolyte trimethylamine N-oxide (TMAO) is established in biochemistry as one of the most effective known stabilizers of macromolecular structures under denaturing stresses [16,17,18]. This function is attributed to its exceptional capacity to organize a dense and tightly bound hydration shell. This fundamental insight has subsequently guided the design of TMAO-derived zwitterionic polymers, which have emerged as a novel class of bioinspired materials distinguished by superhydrophilicity and ultralow fouling characteristics. Notably, recent studies have demonstrated that polymer brushes incorporating the TMAO motif retain robust surface hydration upon exposure to highly concentrated saline environments. This superior salt tolerance arises from the compact dipole and persistent hydration layer of the TMAO zwitterion, which collectively establish an effective barrier against ionic interference. This specific property presents a compelling molecular design strategy to address the critical degradation of fracturing fluid performance when exposed to high-salinity flowback fluid. Recent advances in flowback water hydro-geochemistry have highlighted the potential for recycling treated wastewater in fracturing operations [19,20]. Meanwhile, polymer composites designed for oilfield applications have demonstrated enhanced salinity tolerance and long-term stability under harsh reservoir conditions [21]. However, despite these developments, the specific strategy of TMAO-based molecular interface regulation has not been translated to petroleum engineering, leaving a critical gap in our ability to solve the interfacial stability problems that currently limit the direct reuse of high-salinity flowback fluid.

In this paper, the relationship between the composition of flowback fluid and the resulting fracturing fluid performance using an in situ molecular interface regulation strategy was investigated. Four flowback fluid samples with distinct compositions were collected from four blocks of tight reservoirs. The apparent viscosity, sand-carrying capacity, temperature resistance, and shear resistance of fracturing fluids prepared directly from these flowback fluids were measured. Key parameters of the flowback fluids, including inorganic salts, metal cations, pH, peroxide value, COD, and total organic carbon (TOC), were also analyzed to evaluate their influence on fracturing fluid performance. Based on the molecular interface regulation approach, the TMAO structure was introduced into the polymer to enhance its shielding effect against ions. Molecular dynamics simulations were employed to examine the complexation between metal ions and the cis-hydroxyl groups of guar gum, revealing the mechanism behind crosslinking site deactivation. By establishing a structure–activity model that links flowback fluid composition, interfacial behavior, and engineering performance, this study provides a solution for the direct recycling of flowback fluid without intensive pretreatment. Through molecular design incorporating zwitterionic TMAO groups into the polymer, the resulting HPAMT fracturing fluid exhibits enhanced salt tolerance, enabling direct preparation with untreated high-salinity flowback fluid. This work not only promotes the transition of hydraulic fracturing from a traditional consumptive mode to a circular mode but also offers a technically viable pathway that balances economic benefit and environmental sustainability for unconventional oil and gas development.

## 2. Results and Discussion

### 2.1. Performance Testing of Guar Gum Fracturing Fluid Systems Prepared Directly with Untreated Flowback Fluid

Base fluids of guar gum fracturing fluid were prepared directly using untreated and preliminary preprocessing flowback fluids, and the apparent viscosities were measured (Table 1). For guar gum base fluids, except for Block 1, whether the flowback fluid was pretreated had a relatively small impact on the apparent viscosity of the base fluid. However, when using untreated flowback fluid to prepare the fracturing fluid, incomplete dissolution and precipitation phenomena were observed. It could be explained that certain components in the flowback fluid significantly affected the solubility and stability of guar gum, particularly the high salinity and metal ions in the flowback fluid. These factors might reduce the solubility of the fracturing fluid, thereby affecting its rheological performance and stability.

**Figure 1:**
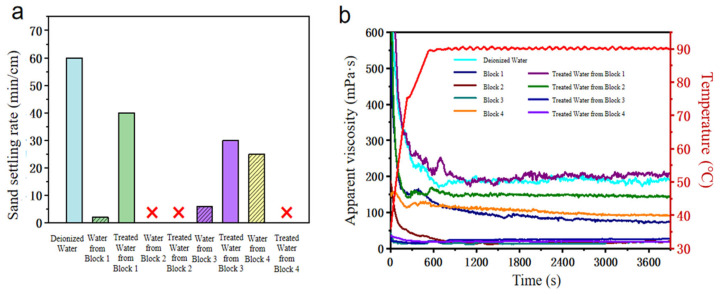
Performance of guar gum fracturing fluid systems prepared directly from flowback fluids (**a**) Sand-carrying performance (**b**) Temperature resistance and shear resistance.

The sand-carrying performance, temperature resistance, and shear resistance of fracturing fluids prepared directly from flowback fluids of four blocks were further analyzed in Figure 1a. Due to the high salinity of the flowback fluids, guar gum failed to form crosslinking effectively, leading to a significant reduction in sand-carrying performance for fracturing fluids prepared with untreated flowback fluid. Specifically, under conditions of 90 °C and shearing at 170 s^−1^ for 1 h, the guar gum fracturing fluid prepared with deionized water maintained an apparent viscosity above 200 mPa·s. Whereas fracturing fluids prepared with flowback fluid showed that multiple systems could not form stable gels. After shearing for 1 h, their viscosities were all below 20 mPa·s (Figure 1b). The high salinity and metal ions in the flowback fluid inhibited the crosslinking of the guar gum system, leading to a significant decline in the rheological performance under high-temperature conditions.

This phenomenon was mainly attributed to the high salinity of the flowback fluid. Inorganic salt components inhibited effective inter-chain crosslinking of guar gum molecules, leading to a significant decrease in polymer chain solubility and structural stability. Compared to fracturing fluid prepared with deionized water, fracturing fluid prepared directly from flowback fluid showed obvious deterioration in both sand-carrying performance and temperature/shear resistance. Especially under high-temperature shearing conditions, the apparent viscosity of the flowback fluid-prepared system decreased significantly, making it difficult to meet the performance requirements for proppant suspension and transport within the formation. The fracture support effectiveness would be weakened, and fracture closure might occur. It was evident that regardless of whether the flowback fluid undergoes preliminary treatments like flocculation and sedimentation, the performance of the fracturing fluid system formed by compounding it with guar gum could not meet the practical needs of field operations, limiting the potential for practical application in fracturing fluid recycling.

### 2.2. Inhibitory Mechanisms of Key Flowback Fluid Components on Fracturing Fluid Performance

The high content of inorganic salt components in flowback fluid was one of the main factors limiting fracturing fluid recycling. According to data in Table 2, the main cations in fracturing flowback fluid were metal cations, with iron (Fe^3+^) and boron (B^3+^) ion content being low. Anions mainly included chloride (Cl^−^), sulfate (SO_4_^2−^), and bicarbonate (HCO_3_^−^). In the preparation of guar gum fracturing fluid, sodium carbonate was typically used to adjust the pH value. HCO_3_^−^ converted to CO_3_^2−^ under alkaline conditions. Since the concentration of CO_3_^2−^ in the flowback fluid was low, the influence of HCO_3_^−^ on guar gum fracturing fluid performance could be neglected.

**Figure 2:**
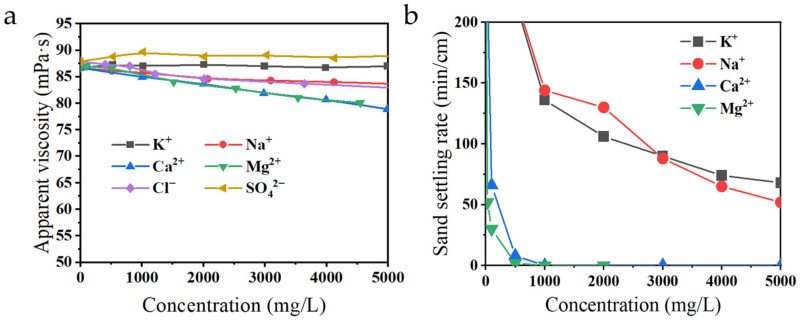
The influence of metal cation content on the performance of guar gum fracturing fluid (**a**) Apparent viscosity changes (**b**) Static sand-carrying performance.

As shown in Figure 2a, the apparent viscosity of guar gum fracturing fluid changed with the increase in ion concentration. When chloride (Cl^−^) and sulfate (SO_4_^2−^) ion concentrations increased to 5000 mg/L, the change in fracturing fluid apparent viscosity was relatively small. It could be explained that guar gum, as a polysaccharide macromolecule, did not carry a positive charge on its polymer chain, and its six-membered ring repeating units possessed high rigidity and steric hindrance, limiting their interaction with anions. However, when the carboxyl groups in guar gum coordinated with metal cations in the flowback fluid, the originally extended polymer chains curled, leading to a decrease in the hydrodynamic volume of polymer, thereby reducing the solution viscosity. Consequently, as the metal cation concentration increased, the apparent viscosity of the fracturing fluid decreased significantly.

Figure 2b indicates that increasing cation concentration significantly reduced the sand-carrying capacity of the fracturing fluid, with Ca^2+^ and Mg^2+^ having a particularly pronounced effect. Polymer dissolution in water was an endothermic process. When water molecules form hydrated bonds with polymer chains, interactions within and between polymer molecules must be overcome. According to Collins’ law of matching water, in aqueous solutions containing metal cations, water molecules tended to bind more strongly to highly hydrated metal cations [22]. Furthermore, the negatively charged parts of water molecules might engage in ion-dipole interactions with cations. Since water was a strong competitive solvent, metal cations could weaken intermolecular interactions between polymers. The presence of metal cations caused many water molecules to detach from polymer chains, inhibiting effective hydration of the polymer chains and preventing their full extension, which directly affected the supporting capacity for quartz sand.

Additionally, since commonly used polymers in fracturing fluids typically contain anionic carboxyl groups, metal ions bind to these carboxyl groups through electrostatic interactions, forming localized crosslinked structures. This process induced curling of the polymer chains and reduced the hydrodynamic volume, thereby weakening the thickening performance. Owing to the high concentration of inorganic salts and multivalent metal ions in flowback fluid, fracturing fluids prepared directly from such sources failed to meet the operational requirements of hydraulic fracturing.

Combining Figure 2a,b, and Hofmeister series analysis, it could be concluded that the influence of metal cations on fracturing fluid performance was dominated by Mg^2+^ and Ca^2+^, whose effects were similar to each other and far exceeded those of Na^+^ and K^+^ [23]. Ca^2+^ and Mg^2+^ in flowback fluid had similar effects, while Na^+^ and K^+^ had similar effects. Since the content of Mg^2+^ and K^+^ in the flowback fluid was relatively low, subsequent research would primarily focus on the influence mechanisms of Ca^2+^ and Na^+^ to improve the performance of fracturing fluids prepared from flowback fluid.

**Figure 3:**
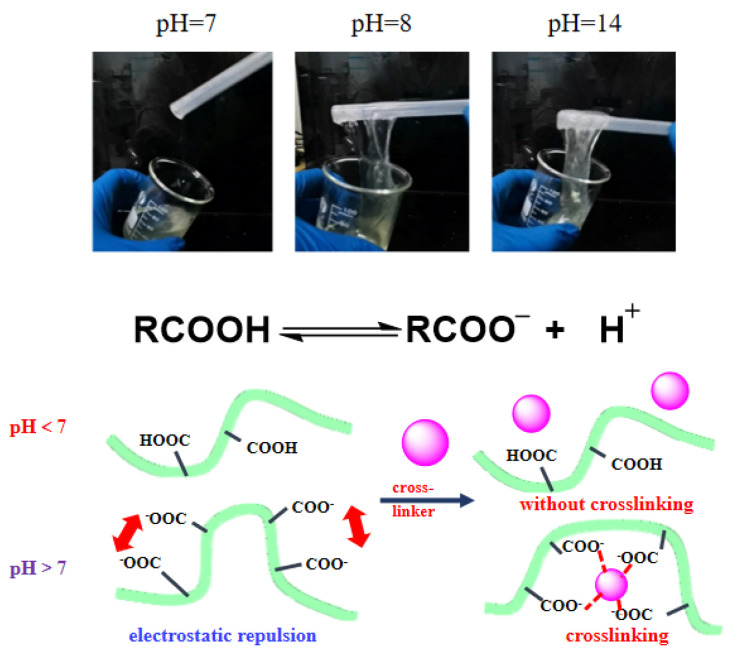
Gel formation of guar gum fracturing fluid system under different pH values.

Combining the results from Table 3 and Figure 3, under acidic conditions, guar gum fracturing fluid could not form crosslinks, while under alkaline conditions, guar gum could form viscoelastic gels, which were crucial for fracturing fluid use. When preparing guar gum fracturing fluid, alkali should be added to adjust the pH value to promote crosslinking. Therefore, the weak alkalinity of flowback fluid had little effect on the pH and crosslinking of guar gum-based fluid, indicating that the pH of flowback fluid was not a major constraining factor for constructing flowback fluid recycling systems.

**Figure 4:**
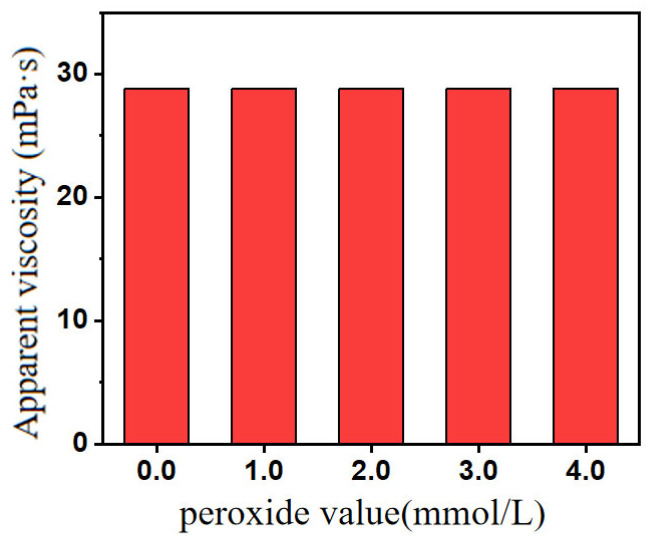
Change in apparent viscosity of base fluid of guar gum fracturing fluid under different peroxide values.

Peroxides (such as potassium persulfate, ammonium persulfate, and so on) were common initiators that could initiate monomer polymerization to form high molecular weight polymers. Figure 4 showed that within the peroxide concentration range of 0 to 4.0 mmol/L, the apparent viscosity of the polymer base fluid for fracturing showed almost no significant change. The degradation of polymers by peroxides occurred when polymer chains absorbed oxygen under high-temperature conditions or were oxidized by peroxides to form peroxide bonds. Due to the instability, peroxide bonds easily decompose, releasing highly oxidative hydroxyl radicals and unstable oxygen radicals. These oxygen radicals continued to attack the polymer backbone, causing polymer chain scission, breaking down into smaller polymer fragments, and eventually degrading into small molecules [24]. However, within the concentration range presented in fracturing flowback fluid, peroxides had almost no significant impact on the performance of the fracturing fluid system.

**Figure 5:**
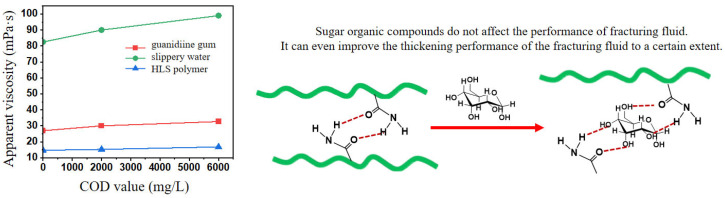
Results of apparent viscosity changes for three types of fracturing base fluids prepared with water of different COD values and a schematic diagram of the action mechanism.

The COD value represented the concentration of reducing substances in water, reflecting the degree of organic pollution. As shown in Figure 5, water samples with higher COD values did not significantly affect fracturing fluid performance. However, when water contained hydroxyl-rich sugar compounds like glucose, the apparent viscosity of the fracturing fluid base fluid increased. The most likely explanation was that hydroxyl groups in glucose formed hydrogen bond interactions with polymer chains, thereby strengthening the hydrogen bond network between polymers and subsequently increasing fracturing fluid viscosity [25].

Under the multiple influences of inorganic salts, high-valent metal ions, peroxides, pH, and COD value, the high concentrations of Ca^2+^ and Na^+^ in flowback fluid were the core factors causing fracturing fluid performance degradation. Therefore, targeted regulation and design for these key ions were crucial. Although metal ion chelating and shielding agents were widely used to improve fracturing fluid performance in high-salinity environments, the effectiveness of using chelating agents to shield calcium ions was very limited. Adding metal chelating agents was an effective way to improve solubility and thickening performance; however, the sand-carrying performance of the fracturing fluid could not be restored completely under the influence of high-valent metal ions. As this approach did not significantly enhance fracturing fluid performance, the effectiveness of flowback fluid recycling was greatly limited. Therefore, further optimizing the shielding effect on metal ions in flowback fluid and enhancing the polymer’s own hydration capability has become a necessary choice for improving the recycling efficiency of fracturing flowback fluid in tight reservoirs.

### 2.3. Interfacial Regulation Mechanism Based on Molecular Design of Salt-Resistance Polymers

**Figure 6:**
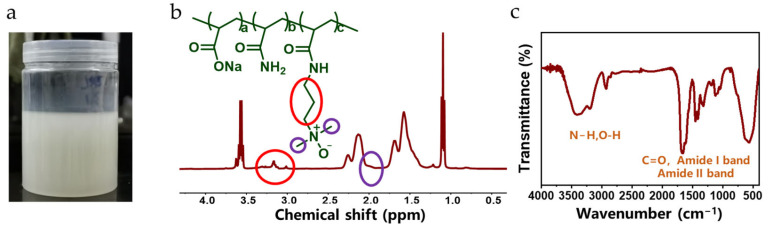
The synthesized HPAMT emulsion. (**a**) Appearance (**b**) ^1^H NMR spectrum (**c**) FTIR spectrum.

The hydration performance of polymers was enhanced by introducing strongly hydrating functional groups onto the polymer chains to counteract the inhibition of fracturing fluid performance by high-valent metal ions. Specifically, by introducing the TMAO group, a novel salt-resistant polymer, HPAMT, was successfully synthesized, as shown in Figure 6a. Figure 6b showed the ^1^H NMR spectrum of HPAMT. Chemical shifts δ = 1.36–1.77 and δ = 2.03–2.34 corresponded to proton peaks of the -CH_2_- and -CH- groups on the HPAMT backbone. δ = 3.01 and δ = 3.17 corresponded to proton peaks of the -CH_2_-CH_2_-CH_2_- group in the TMAO monomer. δ = 1.98 corresponded to proton peaks of the two -CH_3_ groups attached to the nitrogen atom in the TMAO monomer. Figure 6c showed the FTIR spectrum of HPAMT. The broad absorption band observed at 3300–3500 cm^−1^ is assigned to the stretching vibrations of -OH and -NH_2_ groups. The prominent peak at 1660 cm^−1^ is attributed to the C=O stretching vibration (Amide I band) of the acrylamide units. The peaks at 2920 cm^−1^ and 1460 cm^−1^ are assigned to the C–H stretching and –CH_2_– bending vibrations of the polymer backbone, respectively. Additionally, the characteristic absorption band at 1038 cm^−1^ is definitively assigned to the N–O stretching vibration of the trimethylamine N-oxide (TMAO) motif, confirming the successful chemical incorporation of the zwitterionic structure into the HPAMT chain. Experimental results showed that characteristic absorption peaks in the NMR and FTIR spectra were consistent with the structure of HPAMT, and relevant absorption peaks of the TMAO functional group were clearly identified in the spectra, confirming the successful synthesis of HPAMT polymer.

In the zwitterionic structure of TMAO, positive and negative charges were connected by chemical bonds, rendering the molecule overall electrically neutral but exhibiting strong polarity in local regions [26]. This structure endowed TMAO groups with strong hydration ability, effectively binding water molecules and forming a stable hydration layer. Additionally, TMAO groups could repel other contaminants, thereby enhancing interactions between polymer chains and water molecules. While maintaining electrical neutrality, the local polarity of TMAO groups significantly improved the hydration performance of polymer chains and enhanced the competitive ability for water molecules against metal ions, consequently improving polymer stability and performance in high-salinity solutions.

**Figure 7:**
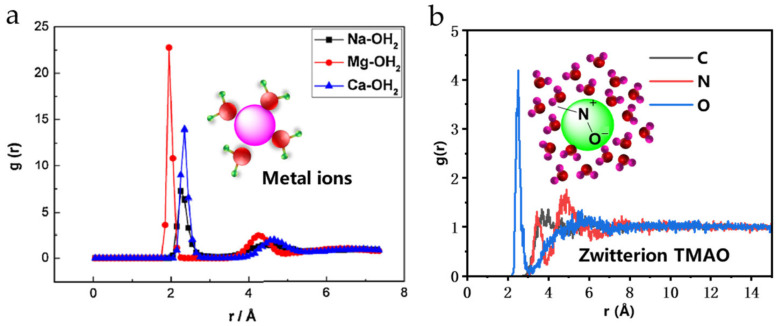
Molecular dynamics simulation results. (**a**) Radial distribution function between metal ions and water molecules (**b**) Radial distribution function between TMAO and water molecules.

As shown in Figure 7 and Table 4, the radial distribution functions between metal ions, TMAO zwitterions, and water molecules in aqueous solution were calculated from molecular dynamics simulations. Simulation results showed that the first hydration shell of TMAO could bind 22.2 water molecules, far exceeding the 2 to 4 water molecules bound by metal ions. Furthermore, TMAO functional groups formed a hydration layer approximately 0.25 nm thick on the polymer chain surface, significantly shielding the adverse effects of metal ions in flowback fluid on fracturing fluid performance, thereby improving polymer stability and hydration performance in high-salinity environments.

**Figure 8:**
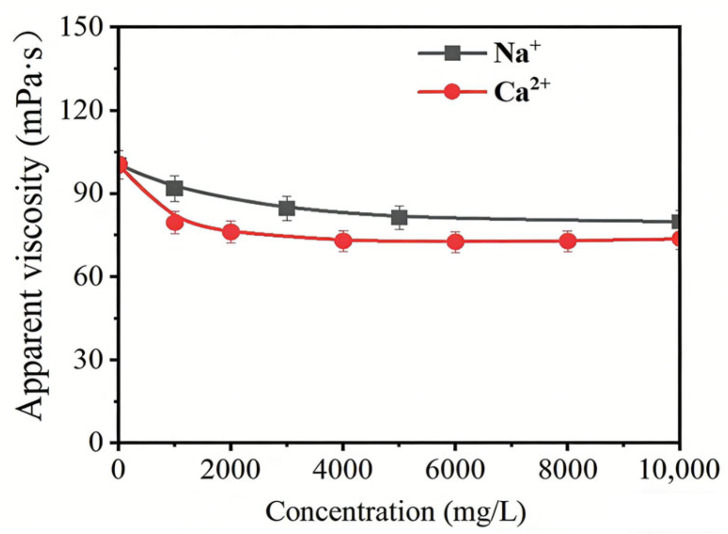
Apparent viscosity change in HPAMT base fluid under different conditions. Na^+^ and Ca^2+^ contents.

Then, the salinity resistance testing of HPAMT was conducted. Introducing the strongly hydrating structure of the TMAO group into the polymer chain not only significantly enhanced the hydration performance of the polymer but also effectively reduced competition from metal ions for polymer-bound water molecules, thereby improving the solubility and thickening performance of the polymer in high-salinity solutions. Consequently, the synthesized HPAMT exhibited excellent salinity resistance. Under conditions where Na^+^ and Ca^2+^ concentrations were gradually increased to 10,000 mg/L, the base fluid viscosity retention of 0.3% HPAMT consistently exceeded 70%, as shown in Figure 8.

The structural origin of the enhanced salt tolerance observed for HPAMT was examined by considering multiple factors. The observed performance gains stemmed from a synergistic combination of the specific TMAO motif and the hybrid zwitterionic–anionic nature of the polymer. Among these, the TMAO motif was identified as the primary contributor to salt tolerance through its unique hydration capacity. Molecular dynamics simulations demonstrated that each TMAO group bound up to 22.2 water molecules and formed a dense hydration layer approximately 0.25 nm thick on the polymer chain surface (Figure 7, Table 4). This robust hydration layer effectively shielded the polymer backbone from ion-induced dehydration and chain collapse. The critical role of such hydration protection was underscored by the performance of conventional guar gum systems, which suffered severe viscosity loss and failed to form stable gels in high-salinity flowback fluids containing elevated Ca^2+^ and Na^+^ concentrations (as shown in Figure 1 and Table 1). In contrast, HPAMT retained more than 70% of its initial viscosity under comparable saline conditions (Figure 8), directly demonstrating the effectiveness of the TMAO-mediated hydration shield. The hybrid zwitterionic–anionic nature of HPAMT played an essential complementary role. The incorporation of anionic carboxyl groups alongside the zwitterionic TMAO moieties enabled efficient coordination with crosslinkers to form a three-dimensional network, providing the structural integrity necessary for subsequent gel formation. Consequently, the TMAO motif dominated salt tolerance through its hydration shield, while the hybrid architecture ensured crosslinking capability. These features established the in situ molecular interface regulation that underpinned the direct recyclability of untreated flowback fluid.

**Figure 9:**
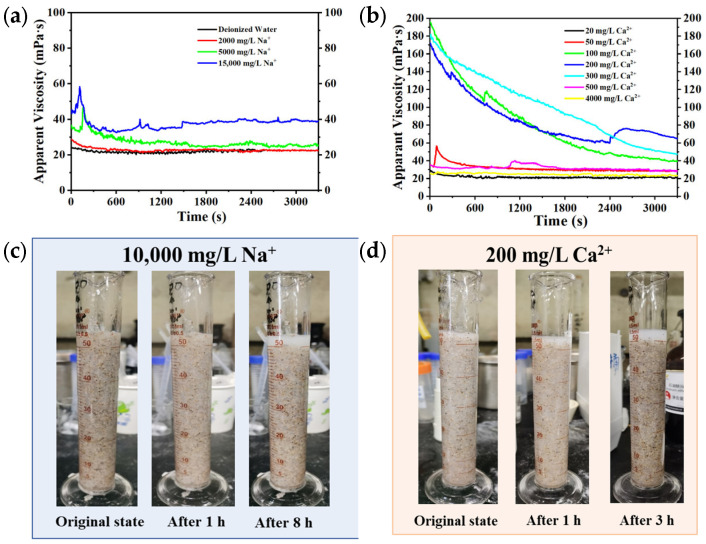
Salinity resistance of HPAMT fracturing fluid (**a**) Rheological curves under different Na^+^ contents (**b**) Rheological curves under different Ca^2+^ contents (**c**) Static sand-carrying photograph under 10,000 mg/L Na^+^ (**d**) Static sand-carrying photograph under 200 mg/L Ca^2+.^

The strong polar dipole structure of the TMAO functional group promoted dipole–dipole interactions within and between polymer chains, thereby affecting polymer chain conformation. With increasing inorganic salt concentration, the strength, temperature resistance, and shear resistance of the HPAMT polymer gel significantly enhanced (as shown in Figure 9a,b). Furthermore, after shearing at 90 °C and 170 s^−1^ for 1 h, the HPAMT fracturing fluid still exhibited good static sand-carrying performance when cooled back to room temperature (Figure 9c). These results indicated that the crosslinking of HPAMT fracturing was relatively uniform, and the formed gel performed well in terms of proppant dispersion and sand-carrying performance. Consequently, the significant enhancement of polymer fracturing fluid salinity resistance and shear resistance by the TMAO zwitterionic group could be demonstrated.

The performance of HPAMT was further contextualized by comparison with representative zwitterionic polymers reported in the literature, as shown in Tables S1 and S2. Conventional sulfobetaine-based polymers, including PSBMA, exhibited the anti-polyelectrolyte effect; however, their coil expansion typically saturated at high ionic strength. These polymers remained vulnerable to divalent cations that disrupted hydration and induced chain collapse [27]. In contrast, HPAMT retained more than 70% of its initial viscosity in flowback fluid containing up to 10,000 mg/L Ca^2+^. At this concentration, many conventional zwitterionic or anionic polymers suffered from precipitation. This resilience was attributed to the exceptionally strong hydration layer formed by TMAO, as demonstrated in previous studies.

A hydrophobically modified zwitterionic polymer designated HPC-5 was developed for ultra-deep well fracturing applications (Table S1). This polymer withstood high salinity (up to 10 × 10^4^ ppm NaCl and CaCl_2_) and elevated temperature (160 °C) [28]. Under the milder testing conditions employed in the present work, with the temperature of 90 °C, HPAMT exhibited comparable viscosity retention and shear stability when prepared directly with untreated flowback fluid. This finding highlighted its suitability for tight reservoir applications. Another system, the rigid-backbone zwitterionic copolymaleimide ZI-PEMA, displayed continuous coil expansion in salt solutions and enhanced divalent salt solubility [29]. Its behavior, however, was examined only in model brine solutions. In contrast, HPAMT was validated in real, untreated flowback fluid containing organic residues and multiple ionic species. Therefore, HPAMT represented a practical solution that combined the molecular-level hydration protection of TMAO with engineering feasibility for direct flowback recycling.

Typically, charged polymers exhibited polyelectrolyte behavior in aqueous solutions. As the inorganic salt concentration increased, the hydrodynamic volume of the polymer decreased. However, HPAMT, as a zwitterionic polymer, exhibited behavior different from traditional charged polymers. With the introduction of inorganic salts, the polymer coil size actually increased. This anti-polyelectrolyte effect was closely related to dipole–dipole pairing interactions between zwitterionic groups. Similar anti-polyelectrolyte behavior, characterized by chain expansion with increasing salt concentration, has been reported in various zwitterionic polymer systems, including copolymaleimides, imidazole-functionalized polymers for high-temperature drilling fluids, and ampholytic terpolymers [30,31]. Before introducing external inorganic salts, dipole–dipole pairing within the polymer chain caused chain contraction. The introduction of inorganic salt ions disrupted the pairing between zwitterionic groups on the polymer chain through electrostatic interactions. This disruption promoted chain extension, increased the hydrodynamic volume, and established an efficient ion shielding mechanism.

### 2.4. Engineering Compatibility Verification of the HPAMT-Enabled Untreated Flowback Fluid Recycling System

**Figure 10:**
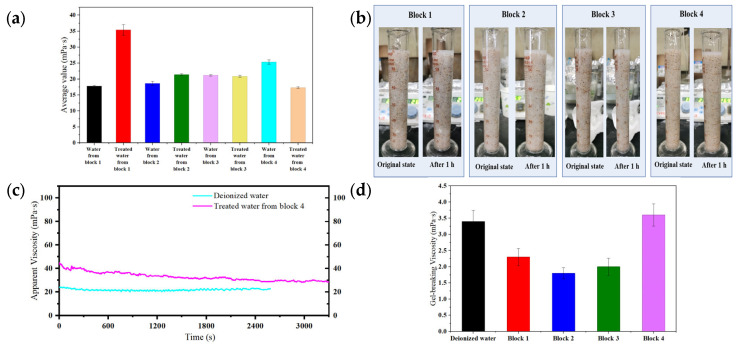
Performance of HPAMT gel fracturing fluid prepared with untreated flowback fluid (**a**) Base fluid apparent viscosity (**b**) Static sand-carrying photographs of HPAMT. (**c**) Temperature resistance and shear resistance (**d**) Gel viscosity after gel-breaking.

Performances of HPAMT fracturing fluid prepared with untreated flowback fluid were tested. As shown in Figure 10a, the base fluid apparent viscosity of HPAMT in flowback fluids from each block was higher than 15 mPa·s, meeting the application requirements for fracturing fluids in tight oil reservoirs. Meanwhile, HPAMT dissolved rapidly within 5 min when prepared with flowback fluid, with no flocculation or precipitation observed, indicating good compatibility between HPAMT and flowback fluid. It was worth noting that untreated flowback fluid contained a certain concentration of suspended solids. These particles were considered to contribute to the internal structural strength of the gel, particularly enhancing the low-shear viscosity through particle-polymer interactions. The zwitterionic motifs in HPAMT may have further stabilized these interfaces, ensuring that the presence of solids did not lead to detrimental phase separation. This observation supported the feasibility of the pretreatment-free recycling strategy. Figure 10b showed the sand-carrying performance of HPAMT fracturing fluids prepared directly from untreated flowback fluids of each block. Results showed that fracturing fluids prepared with flowback fluid possessed excellent static sand-carrying performance, with static sand-carrying times all exceeding 1.0 h/cm. The experimental data were compared with the theoretical Stokes’ settling model to further understand the proppant suspension mechanism. For 20/40 mesh proppant, the calculated theoretical settling velocity in a 200 mPa·s Newtonian fluid was significantly faster than the experimental observations. This deviation indicated that the HPAMT gel provided structural support beyond simple viscous drag, likely due to the elastic network formed by the zwitterionic TMAO side groups, which effectively resisted proppant sedimentation under static conditions.

**Figure 11:**
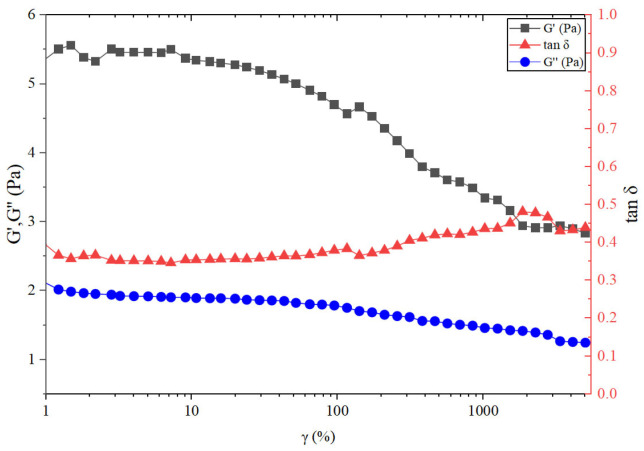
Relationship between viscoelastic moduli and strain in the HPAMT gel fracturing fluid.

The temperature resistance, shear resistance, and broken gel viscosity of HPAMT fracturing fluid prepared with untreated flowback fluid are shown in Figure 10c,d. The HPAMT fracturing fluid exhibited excellent temperature resistance and shear resistance. During the heating stage from 25 °C to 90 °C, the viscosity curves were smooth without abrupt changes, mainly attributed to the effective inhibition by the TMAO hydration layer of polymer chain dehydration-induced coiling under high-temperature conditions. The smooth viscosity profile of HPAMT gel fracturing fluid under continuous shear indirectly reflected a high degree of structural integrity, preventing local stress concentration and premature network failure. The viscosity reduction observed at 90 °C was interpreted as a synergistic result of multiple factors. Thermal dissociation of coordination bonds between HPAMT and the crosslinker occurred, where increased thermal energy shifted the dynamic equilibrium toward a dissociated state. The enhanced molecular mobility of the polymer chains led to a reduction in entanglement density through reptation dynamics. Additionally, an intrinsic decrease in zero-shear viscosity (η_0_) followed an Arrhenius-type relationship with temperature. Notably, the TMAO motifs established a robust “hydration atmosphere” that acted as a thermal buffer, stabilizing the polymer backbone and delaying the catastrophic breakdown of these physical and chemical networks. As a result, the HPAMT fracturing fluid retained more than 90% of its initial viscosity after 60 min of shear at 90 °C, outperforming conventional fracturing fluid systems that lacked. For fracturing fluids prepared with untreated flowback fluids, after shearing for 1 h, the broken gel viscosity consistently remained below 5 mPa·s. The electrically neutral nature of the TMAO group effectively reduced adsorption within the formation, indicating that the low viscosity characteristic of HPAMT was beneficial to mitigate damage caused by liquid phase retention. Simultaneously, the electrically neutral design inhibited clay swelling, significantly reducing the comprehensive permeability damage rate. As shown in Figure 11, the elastic modulus G′ of the HPAMT consistently exceeded the viscous modulus G″, with tan*δ* values below 0.5. This behavior confirmed the formation of a stable, elastic-dominated gel network. Moreover, the relatively low frequency dependence of G′ indicated a homogeneous crosslinking structure without significant relaxation processes. This provided direct experimental evidence for the uniform gel network previously inferred from the smooth viscosity plateau in Figure 10c.

HPAMT demonstrated broad compatibility with various untreated flowback fluids. The base fluid viscosities were greater than 10 mPa·s in all cases, and they could directly form gels without pre-treatment. The HPMT fracturing fluid exhibited static sand-carrying time exceeding 60 min/cm, thereby overcoming the conventional reliance of fracturing flowback fluid recycling on intensive pretreatment. Economic evaluation indicated that the direct reuse scheme of HPAMT eliminated steps like flocculation, filtration, and desalination, compressing treatment costs to 30% of traditional processes. Taking single-well consumption of 5000 m^3^ of flowback fluid as an example, Pre-treatment-free recycling of flowback fluid could save approximately 1.2 million RMB in freshwater procurement and wastewater treatment costs. This estimate was based on current field practices in the Daqing Oilfield, where the reuse of flowback fluid eliminated the need for advanced treatment and discharge, saving 210 RMB per cubic meter, reduced storage-related expenses by 20 RMB per cubic meter, and substituted freshwater typically used for fracturing fluid preparation, saving an additional 10 RMB per cubic meter. These combined savings amounted to 240 RMB per cubic meter of flowback fluid reused. While these figures reflected local conditions at the time of analysis, actual savings may vary depending on site-specific factors and regulatory requirements. The results demonstrated significant economic benefits alongside good environmental performance and engineering feasibility.

By introducing zwitterionic functional side groups onto the polymer chain, HPAMT gel exhibited properties different from conventional zwitterionic polymers. Dipole–dipole pairing between TMAO groups formed interactions within and between polymer chains. However, unlike pure zwitterionic polymers, the polymer chain of HPAMT also contained anionic carboxyl groups, imparting additional net charge to the polymer chain [32,33]. Under application conditions of weakly alkaline, the anionic carboxyl groups carried a negative charge, which might disrupt the dipole–dipole pairing between TMAO zwitterions, instead forming interactions between carboxylate anions and the zwitterions. Nevertheless, due to factors like the steric hindrance of the carboxylate anions, segments of the polymer chain containing zwitterionic side groups could still form dipole–dipole pairs, causing a certain degree of conformational coiling in the polymer chain. This curling effect could influence the strength of the crosslinking network between carboxylate anions and the crosslinking agent. The structural recovery of the HPAMT gel was critical for its performance in tight reservoirs. Given that the crosslinking was based on reversible coordination and dipole–dipole interactions, the HPAMT system exhibited significant thixotropic characteristics. Upon cessation of high-shear pumping, the dissociated polymer segments could rapidly reorganize into a three-dimensional viscoelastic network. This rapid structural recovery was indirectly evidenced by the sustained sand-carrying capacity observed after long-term shear (Figure 9c), ensuring that the proppant remained uniformly distributed within the hydraulic fractures.

**Figure 12:**
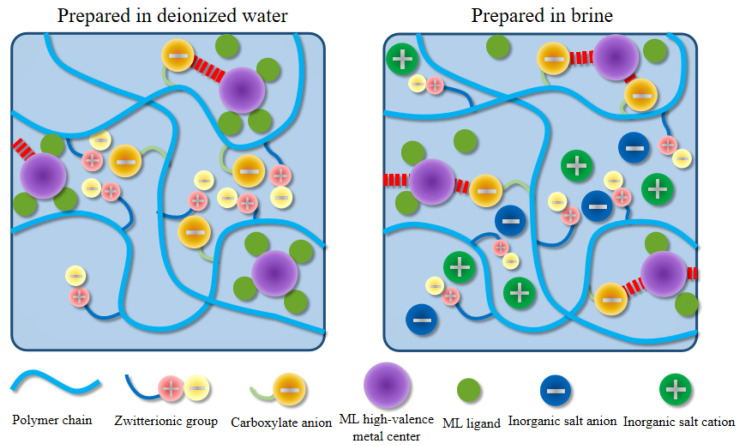
Schematic diagram of salinity resistance and salt-thickening mechanism of HPAMT fracturing fluid.

When external inorganic salts or high-valent metal ions were present, the inorganic salts replaced the role of carboxylate anions in disrupting the dipole–dipole pairing of zwitterionic groups. Simultaneously, influenced by the anti-polyelectrolyte effect, partially coiled polymer chains extended. The encapsulated carboxylate anions were exposed, and the crosslinking between carboxylate anions and the coordination crosslinker was better. Figure 12 illustrates the excellent performance of the developed HPAMT polymer in terms of salinity resistance and shear resistance, along with the good compatibility with flowback fluid.

## 3. Conclusions

Metal ions such as Ca^2+^ and Na^+^ significantly degrade the performance of fracturing fluids prepared directly with untreated fracturing flowback fluid through dehydration effects and electrostatic complexation. Under the high-salinity conditions typical of tight reservoirs, conventional fracturing fluid systems suffer viscosity losses exceeding 60% and lose their sand-carrying capacity. Based on a molecular interface regulation strategy, this study incorporated a zwitterionic TMAO group into polyacrylamide to mitigate competitive hydration, the process by which metal ions strip water molecules from polymers, inducing chain coiling and hydrodynamic radius compression. Then, a novel salt-tolerant and shear-resistant polymer (HPAMT) was developed. The TMAO zwitterionic moiety can bind up to 22.2 water molecules, forming a dense hydration layer that markedly improves viscosity retention. Compared with conventional polymers, HPAMT achieves a viscosity retention increase of over 50%. Owing to its anti-polyelectrolyte effect, HPAMT exhibits salt-thickening behavior. In fracturing fluids prepared directly from flowback fluid, HPAMT provides a static proppant suspension time exceeding 60 min/cm. After shearing at 90 °C and 170 s^−1^ for 60 min, the viscosity retention remains above 90%, and the broken gel viscosity is below 5 mPa·s. HPAMT fracturing demonstrates excellent compatibility with untreated flowback fluid and meets all key performance indicators without intensive pretreatment, confirming the feasibility of direct flowback fluid reuse in tight reservoirs. This work establishes a quantitative structure-activity relationship model between flowback fluid composition and fracturing fluid performance, providing theoretical support for a pretreatment-free recycling strategy. The findings promote the transition of fracturing operations toward a circular water-use model, contributing to both economic and environmental sustainability in unconventional resource development.

## 4. Materials and Methods

### 4.1. Materials and Instruments

The control fracturing fluid system used in this study was a guar gum fracturing fluid, whose formulation includes 0.3% guar gum, 0.1% sodium carbonate, 0.2% alkylbenzene sulfonate, and 0.2% crosslinker. The hydrolyzed polyacrylamide grafted with trimethylamine N-oxide polymer (HPAMT) used in this study was an emulsion-type polymer synthesized through inverse emulsion polymerization. In the fracturing fluid formulation, 0.3 wt% of a crosslinker was added to the HPAMT-based system. The flowback fluids used were selected from four blocks in the tight reservoirs. Flowback fluids before and after preliminary flocculation and filtration treatment were used to prepare fracturing fluids, and the performances were tested.

The instruments used in this study are shown as follows. ZNN-D6B Six-speed Rotational Viscometer, Qingdao Hengtaida Mechanical and Electrical Equipment Co., Ltd., Qingdao, China; HAAKE RS6000 Rheometer, Thermo Fisher Scientific, Karlsruhe, Germany; Inductively Coupled Plasma Atomic Emission Spectrometer iCAP 7400, Thermo Fisher Scientific, Bremen, Germany; Nuclear Magnetic Resonance Spectrometer AVANCE III 400, Bruker, Rheinstetten, Germany; Fourier Transform Infrared Spectrometer Bruker TENSOR II, Bruker, Ettlingen, Germany.

### 4.2. Experimental Methods

#### 4.2.1. Preparation of Guar Gum Fracturing Fluid System

The commonly used fracturing fluid system in the oilfield was the guar gum fracturing fluid system. According to the above formulation in Section 2.1, guar gum, sodium carbonate, alkylbenzene sulfonate, and crosslinker were added to deionized water by mass ratio, stirred thoroughly until completely dissolved, ensuring a uniform and stable solution to form the desired fracturing fluid. For testing the impact of different flowback fluids on fracturing fluid performance, flowback fluid samples were collected from four blocks in the tight reservoirs of the Daqing oilfield. The four flowback fluid samples were then subjected to preliminary flocculation and filtration treatment to remove suspended solid impurities, ensuring sample impurities would not interfere with subsequent performance tests.

#### 4.2.2. Characterization of Properties of Fracturing Fluid

After preparing the guar gum fracturing fluid, the apparent viscosity was measured using a ZNN-D6B six-speed rotational viscometer (Qingdao Hengtaida Mechanical and Electrical Equipment Co., Ltd., Qingdao, China) at a shear rate of 170 s^−1^, with a test temperature of 25 °C. During measurement, it was ensured that the viscometer spindle was thoroughly cleaned to avoid residual effects from previous experiments on test results.

The static sand-carrying performance of the fracturing fluid was tested as follows. Firstly, a 50 mL fracturing fluid sample was formulated as specified. Then, 20/40 mesh quartz sand proppant was added at a sand-carrying ratio of 30% (sand mass accounting for 30% of the total slurry mass) and thoroughly stirred to achieve complete dispersion within the fluid. Immediately afterwards, the fracturing fluid with sands was poured into a measuring cylinder. After standing for a certain time, the settling situation of the quartz sand was observed, and the settling rate was recorded. The settling rate was expressed in the dimension of min/cm, representing the time taken for quartz sand to settle 1 cm.

When the complete settling time was less than 1 h, the time for complete settling (*t*) and the height *h* of the supernatant in the cylinder after complete settling were recorded. The Sand settling rate, *S*_r,_ was calculated using the following equation [34].

*S*_r_ = *t/h* (1)

where the dimensions of *t* and *h* were minutes and centimeters, respectively.

When the complete settling time exceeded 1 h, the height *h* of the clear supernatant in the cylinder was recorded after 1 h of static placement, representing the proppant settlement distance within the first hour. The Sand settling rate, *S_r_*_,_ was calculated using the following equation.

*S*_r_ = 60/*h* (2)

After fracturing fluid gel formation, the rheological properties were tested using a HAAKE RS6000 rheometer (Thermo Fisher Scientific, Karlsruhe, Germany). The fracturing fluid gel was subjected to continuous shearing at 90 °C and a shear rate of 170 s^−1^ for 1 h, and the apparent viscosity was measured throughout the test to obtain the apparent viscosity change curve of the fracturing fluid gel. During measurement, the shear rate was kept constant at 170 s^−1^. The experiment consisted of heating and constant temperature stages. The heating process was divided into two stages, including the first stage where the temperature varied from room temperature to 75 °C at a rate of 10 °C/min, and the temperature of the second stage changed from 75 °C to 90 °C at a rate of 3.6 °C/min. After reaching 90 °C, the constant temperature stage began, and stable shearing continued for 60 min, after which testing stopped and cooled. The instrument recorded the apparent viscosity and corresponding temperature every 5 s. Finally, rheological curves of the fracturing fluid gel were plotted from the test data to characterize the temperature resistance and shear resistance of fracturing fluids prepared with flowback fluid.

#### 4.2.3. Determination of the Basic Properties of Flowback Fluids

Multiple analytical methods were employed to characterize the flowback fluid components. Inductively Coupled Plasma Atomic Emission Spectroscopy (ICP-AES) was used to determine cation content in different flowback fluids. Ion chromatography was used to determine chloride (Cl^−^) and sulfate (SO_4_^2−^) ion content. Moreover, acid-base titration was used to determine the ion content of bicarbonate (HCO_3_^−^), carbonate (CO_3_^2−^), and hydroxide (OH^−^).

The pH of flowback fluids was measured using a pH meter. Before measurement, the pH meter probe was cleaned with deionized water and wiped dry. The probe was then inserted into the test liquid, and the reading of the pH value was recorded after stabilization.

Sodium thiosulfate redox titration was used to determine peroxide content in flowback fluids. Firstly, a measured volume of water sample was pipetted into an iodine flask. 50.0 mL of glacial acetic acid was added and mixed, followed by 1.0 mL of saturated potassium iodide solution and mixing. The iodine flask was sealed and placed in the dark for 10.0 min. Subsequently, the flask was opened, rinsed inside with a small amount of deionized water, and immediately titrated with standardized sodium thiosulfate solution. When the solution turned pale yellow, starch indicator was added, and titration continued until the solution turned from pale yellow to colorless. Deionized water was used as a control group, titrated following the same procedure. The peroxide content in the flowback fluid (expressed as peroxide molar concentration) was calculated as half the moles of sodium thiosulfate standard solution consumed divided by the volume of water sample taken, and the dimension was defined as mmol/L. The potassium dichromate method was used to determine the COD value of the fracturing flowback fluid for further assessment of its organic content.

#### 4.2.4. Synthesis, Preparation, and Performance Characterization of HPAMT

The novel salt-resistant and shear-resistant polymer HPAMT was synthesized through free-radical inverse-emulsion copolymerization of acrylamide with a zwitterionic monomer containing a TMAO group. The oil phase was prepared by dissolving the lipophilic surfactant Span 80 in #5 white oil, while the aqueous phase was prepared by dissolving the monomers in deionized water, with the pH adjusted to 6–7. After high-shear emulsification for 10–30 min to form a stable water-in-oil emulsion, the system was purged with inert gas for 30–60 min to remove dissolved oxygen. The polymerization was then initiated by a redox system under steady stirring at 200–400 rpm, with the temperature controlled at 10–60 °C for 4–6 h. For direct field application, Tween 80 was employed as a phase-inversion agent to enhance the dissolution rate of the emulsion in water. For laboratory characterization, the polymer was purified by breaking the emulsion with excess acetone, followed by multiple washing cycles with anhydrous ethanol and vacuum drying to remove any residual oil or surfactants prior to. The structure of HPAMT was characterized using Nuclear Magnetic Resonance (NMR) spectroscopy and Fourier Transform Infrared (FTIR) spectroscopy.

Combined with molecular dynamics simulation, the radial distribution functions between metal ions and the zwitterionic TMAO, as well as water molecules, were calculated, further analyzing the structural characteristics in aqueous solution. The molecular dynamics simulations were performed using the Materials Studio 2020 software package. Individual species, including Na^+^, Ca^2+^, Mg^2+^, TMAO, and H_2_O, were constructed, followed by the creation of an amorphous cell containing 504 water molecules and a single target molecule to simulate an infinitely dilute environment. Energy minimization was conducted using the Geometry Optimization task within the Forcite module, after which a 1000 ps production run was performed in the NPT ensemble to ensure stable equilibrium. Radial distribution functions between the target species and the oxygen atoms of water were subsequently analyzed to determine hydration shell structure and bound water numbers.

Solutions of 0.3% HPAMT polymer were prepared using sodium chloride (NaCl) and calcium chloride (CaCl_2_) solutions containing varying concentrations of Na^+^ and Ca^2+^, respectively, and their apparent viscosities were measured. Subsequently, 0.3% HPAMT was mixed with 0.3% crosslinker to form a gel, and the temperature resistance and shear resistance were further measured under different salinity conditions.

Further tests were conducted to evaluate the performance of fracturing fluid directly compounded by synthesized HPAMT polymer and the untreated flowback fluid intended for recycling. Base fluids containing 0.3% HPAMT were formulated using flowback fluid from each block, and their viscosities were measured. Gel fracturing fluids were then prepared directly from these base fluids with the addition of 0.3% crosslinker, and the temperature resistance, shear resistance, and sand-carrying performance were assessed. 0.05% potassium persulfate was added to a sample prepared directly from flowback fluid and placed in a 65 °C water bath for 12 h to characterize the gel-breaking performance of the HPAMT-based fracturing fluid. The viscosity of the broken gel was subsequently measured using a capillary viscometer.

## Figures and Tables

**Table 1:** Fracturing flowback fluid samples and viscosity of prepared guar gum fracturing fluids.

Number	Flowback Fluid Block	Apparent Viscosity of Guar Gum Base Fluid/(mPa·s)
Deionized Water	-	30.0
Flowback Fluid 1	Block 1	27.3
Flowback Fluid 2	Block 1 (Treated)	27.9
Flowback Fluid 3	Block 2	0.9
Flowback Fluid 4	Block 2 (Treated)	0.9
Flowback Fluid 5	Block 3	6.6
Flowback Fluid 6	Block 3 (Treated)	29.1
Flowback Fluid 7	Block 4	26.7
Flowback Fluid 8	Block 4 (Treated)	25.8

**Table 2:** Ionic components and concentrations of various fracturing flowback fluids/(mg/L).

Flowback Fluid Number	K^+^	Na^+^	Ca^2+^	Mg^2+^	Fe^3+^	B^3+^	Cl^–^	SO_4_^2–^	HCO_3_^–^	CO_3_^2–^
1	27.8	607.4	5.5	0.0	0.0	0.0	550.9	296.7	376.8	13.3
2	17.7	162.4	38.7	11.4	0.0	0.0	0.0	6.1	360.8	13.6
3	7.5	1289.6	29.4	3.2	5.5	5.6	1074.0	7.0	1426.2	0.0
4	5.3	885.4	79.7	30.8	0.0	1.0	762.0	562.0	441.3	0.0
5	18.3	1496.1	17.5	5.7	0.0	17.5	578.0	16.5	2807.6	0.0
6	15.8	1420.1	8.6	1.7	0.0	5.6	556.5	22.5	2665.9	0.0
7	8.9	845.8	168.7	2.9	2.3	6.0	863.0	827.0	1302.4	0.0
8	17.9	1056.2	403.5	9.7	0.5	11.0	1670.0	1324.0	319.3	0.0

**Table 3:** Properties of Fracturing Flowback Fluids.

Flowback Fluid Number	pH	Peroxide Value/(mmol/L)	COD
1	8.3	2.2	334
2	8.4	0.6	12
3	7.7	1.3	59
4	7.9	2.3	192
5	7.5	3.5	430
6	7.9	1.9	170
7	8.0	1.9	1950
8	6.3	0.6	5690

**Table 4:** First Hydration Shell Radius and Number of Bound Water Molecules for Metal Ions and TMAO.

Ion or Group	First Hydration Shell/nm	Number of Bound Water Molecules
Na^+^	0.23	2.6
Ca^2+^	0.24	3.6
TMAO	0.25	22.2

## Data Availability

Data is contained within the article or Supplementary Materials. The original contributions presented in this study are included in the article/Supplementary Materials. Further inquiries can be directed to the corresponding author.

## Supplementary Materials

The following supporting information can be downloaded at: https://www.mdpi.com/article/10.3390/gels12020178/s1, Table S1: Performance comparison between HPAMT and HPC-5 zwitterionic polymer systems; Table S2: Comparison of properties of representative zwitterionic polymer systems.
