# Supplementary material for: Zwitterionic Polymer Gel Fracturing Fluid with Molecular Interface Regulation for Pretreatment-Free Flowback Recycling
Source: Gels. 2026 Feb 19;12(2):178. doi: 10.3390/gels12020178 (PMC12940335; doi:10.3390/gels12020178)
Supplement: Supplementary file 1 [file gels-12-00178-s001.zip › gels-4139067-supplementary.pdf]

As shown in Table S1, we have compared HPAMT with the recently reported HPC-5 zwitterionic hydrophobic polymer fracturing fluid system developed for ultra-deep high-temperature wells in the Tarim Basin [Jian et al., 2024 Colloid Polym Sci]. Both polymers are zwitterionic synthetic polymers designed for hydraulic fracturing applications using high-salinity water sources. This comparison demonstrates that HPAMT exhibits comparable or superior salt tolerance and calcium ion resistance to HPC-5, while meeting the specific requirements for direct recycling of untreated flowback fluid in tight reservoirs. The TMAO-induced hydration layer in HPAMT provides effective shielding against ion-induced chain coiling, analogous to the hydrophobic association mechanism in HPC-5.

Table S1. Performance comparison between HPAMT and HPC-5 zwitterionic polymer systems

	HPAMT (This Work)	HPC-5 [Jian et al., 2024]
Polymer type	TMAO-grafted hydrolyzed polyacrylamide	AM/AA/DMC/AMPS/hydrophobic monomer copolymer
Salt tolerance	>70% viscosity retention at 10,000 mg/L NaCl and $CaCl_2$	Maintains high viscosity at 10×10^4 ppm NaCl and $CaCl_2$
Ca^{2+} resistance	>70% viscosity retention at 5,000 mg/L Ca^{2+}	Comparable high values at high $CaCl_2$ concentration
Thermal/shear stability	>90% viscosity retention after 60 min at 90 °C, 170 s^{-1}	Exceptional temperature resistance up to 160 °C for the gel
Application context	Tight reservoir fracturing with untreated flowback fluid	Ultra-deep well fracturing with reservoir brine

As shown in Table S2, HPAMT exhibits comparable or superior salt tolerance and Ca^{2+} resistance to recently developed zwitterionic polymers, while maintaining excellent thermal and shear stability under conditions relevant to tight reservoir fracturing. Notably, HPAMT has been validated in untreated, complex flowback fluid containing multiple interfering ions and organic residues, whereas the literature

systems were typically evaluated in simple model brine solutions. This practical validation further highlights the robustness of the TMAO-based molecular design.

Table S2 Comparison of properties of representative zwitterionic polymer systems.

Polymer	Polymer Type / Key Feature	Salt Tolerance	Ca^{2+} Resistance	Shear Stability	Application Context	Source
pTMAO	TMAO polymer brush; minimal dipole length (N^+–O^-).	Superior resistance; surface hydration only moderately reduced even in seawater.	Exhibited weaker interaction between O^- and metal cations.	Excellent stability against protein adsorption in seawater.	Surface chemistry; nonfouling coatings in marine environments.	[16]
PTMAO	TMAO-derived polymer; compact dipole with no spacer between charges.	Superior hydration resilience in high-salinity environments.	Not directly tested; while mechanism suggested strong resistance to cation disruption.	Not reported in context of rheology.	Fundamental material science; ultralow fouling surfaces.	[18]
HPC-5	Hydrophobically modified zwitterionic polymer for fracturing.	Excellent.	Excellent (Stable up to 10×10^4 ppm $CaCl_2$).	Excellent (160 °C, 170 s^{-1} for 60 min, >80% retention).	Ultra-deep well fracturing fluid.	[28]
ZI-PEMA	Zwitterionic copolymaleimide with a rigid backbone.	Ultra-high (Continuous viscosity increase up to saturated NaCl).	Good (Enhanced $CaBr_2$ solubility; no precipitation reported).	Not reported.	High-density brines; industrial applications.	[29]
	TMAO-grafted hydrolyzed polyacrylamide.	High	High (>70% viscosity retention at 10,000 mg/L Ca^{2+}).	Excellent (90 °C, 170 s^{-1} for 60 min, >90% retention).	Direct recycling of untreated fracturing flowback fluid.	This work

The following references cited in the revised main manuscript are used to improve this table.

[16] Huang, H.; Zhang, C.; Crisci, R.; Lu, T.; Chen, Z. Strong surface hydration and salt resistant mechanism of a new nonfouling zwitterionic polymer based on protein stabilizer TMAO. Journal of the American Chemical Society, 2021,143 (40).

[18] Li, B.; Jain, P.; Ma, J.; Smith, J. K.; Yuan, Z.; Hung, H.-C.; He, Y.; Lin, X.; Wu, K.; Jiang, S.; et al. Trimethylamine N-oxide–derived zwitterionic polymers: A new class of ultralow fouling bioinspired materials. Science Advances, 2019, 5 (6).

[28] Jian, C.; Yu, Y.; Yu, D.; Chen, P.; Yan, J.; Chen, X. Synthesis and Evaluation of Salt Tolerant Delayed-Crosslinking Fracturing Fluid System in Ultra-Deep High Temperature Wells. Colloid and Polymer Science 2024, 302, 1591–1601.

[29] Aitipamula, S.; Hadia, N. J.; Vasantha, V. A.; Parthiban, A. An Exceptionally Salt Tolerant Copoly (Maleimide Sulfobetaine) - Structural Requirements for Ultra-Salt Tolerance. Macromolecular Rapid Communications 2024, 45 (23).
